# Leaving negative ancestors behind

**DOI:** 10.7554/eLife.20061

**Published:** 2016-08-31

**Authors:** Sergio A Muñoz-Gómez, Andrew J Roger

**Affiliations:** Centre for Comparative Genomics and Evolutionary Bioinformatics, CIFAR Program in Integrated Microbial Biodiversity and the Department of Biochemistry and Molecular Biology, Dalhousie University, Halifax, Canadasergio.munoz@dal.ca; Centre for Comparative Genomics and Evolutionary Bioinformatics, CIFAR Program in Integrated Microbial Biodiversity and the Department of Biochemistry and Molecular Biology, Dalhousie University, Halifax, CanadaAndrew.Roger@Dal.Ca

**Keywords:** Negativicutes, Halanaerobiales, Firmicutes, phylogenomics, None

## Abstract

Bacteria with a single cell membrane have evolved from ancestors with two membranes on multiple occasions within the Firmicutes phylum.

**Related research article** Antunes LCS, Poppleton D, Kling A, Criscuolo A, Dupuy B, Brochier-Armanet C, Beloin C, Gribaldo S. 2016. Phylogenomic analysis supports the ancestral presence of LPS-outer membranes in the firmicutes. *eLife* **5**:e14589. doi: 10.7554/eLife.14589

For more than a century bacteriologists have used the Gram stain reaction to classify bacteria. The Gram stain is a violet-colored dye that is retained by Gram-positive bacteria but not by Gram-negative bacteria. These different reactions to the stain reflect fundamental differences in the cell envelopes of these bacteria: Gram-positive bacteria usually have a single cell membrane that is encased by a thick wall made of a polymer called peptidoglycan, whereas Gram-negative bacteria tend to have two membranes with a thin wall of peptidoglycan sandwiched between them.

The tree of life contains about 30 bacterial phyla, but only three of them contain bacteria that are surrounded by a single cell membrane, which are also known as “monoderms”. The remaining phyla contain bacteria with two cell membranes, and most of these “diderms” have large molecules called lipopolysaccharides (LPS) in their outer membranes. However, at least two phyla comprise diderms that do not have LPS.

The evolutionary relationships between monoderms and diderms have remained uncertain for many years. It is generally thought that the monodermic cell plan evolved from the more complex didermic cell plan in a single simplification event (see, for example, Cavalier-Smith, 2006). However, it is possible that diderms could have evolved from monoderms (Dawes, 1981; Tocheva, 2011). Now, in eLife, Simonetta Gribaldo of the Institut Pasteur and co-workers – including Luísa Antunes and Daniel Poppleton as joint first authors – report that monodermic bacteria evolved from ancestral didermic bacteria not once but multiple times by losing the outer membrane from their cell envelopes (Antunes et al., 2016).

Antunes et al. focused on the Firmicutes, a phylum that contains a mixture of monoderms and diderms. By analyzing the genomes of more than 200 members of the phylum, they showed that the two didermic groups – the Negativicutes and the Halanaerobiales – are not each other's closest relatives and are, instead, more closely related to one or more of the monodermic groups. Furthermore, they demonstrate that the biosynthetic machinery for synthesizing their LPS has not been transferred between them nor acquired from elsewhere. Instead, the outer membrane of the didermic firmicutes appears to have been inherited vertically from a distant ancestor. These results suggest that the monodermic firmicutes evolved at least five times from an ancestral and more complex didermic cell plan (Figure 1).

**Figure 1.**
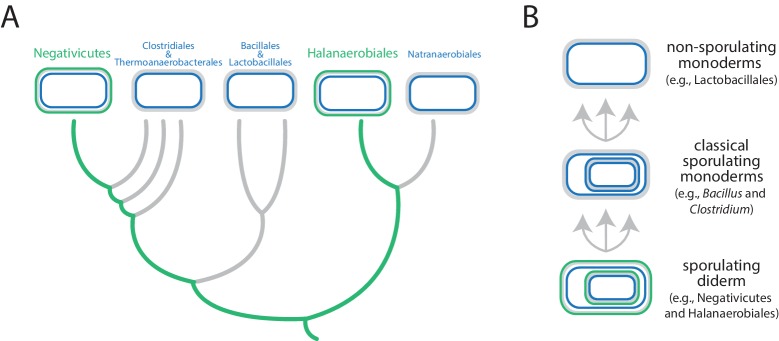
Evolution of the Firmicutes phylum. (**A**) Didermic firmicutes have a cytoplasmic membrane (shown in blue), a peptidoglycan cell wall (gray) and an outer membrane (green), whereas monodermic firmicutes have a cytoplasmic membrane and a peptidoglycan cell wall, but no outer membrane. Antunes et al. show that the ancestral didermic cell plan of the Firmicutes phylum has been lost at least five times. Most lineages lost their outer membranes to become monoderms (thick gray lines), but the Negativicutes and the Halanaerobiales retained the ancestral didermic cell plan (thick green lines). (**B**) Major transitions between bacterial cell plans within the Firmicutes phylum. Ancestral sporulating diderms (similar to the Negativicutes and the Halanaerobiales) convergently gave rise to classical sporulating monoderms (e.g., *Bacillus* and *Clostridium*), which lost the capacity to form endospores in some linages (e.g., *Lactobacillus*). Endospores are shown as cells within cells.

Comparative analyses of the genomes of Negativicutes and Halanaerobiales also allowed Antunes et al. to make inferences about the nature and evolution of their didermic envelopes. Notably, and unusually, most of the genes required for the biogenesis of the outer membrane clustered in a large genomic region in both groups. Moreover, these two groups have envelope appendages (such as flagella and pili) that resemble the envelope appendages of other diderms (in other phyla) more than they resemble those of their close monodermic relatives. Finally, didermic firmicutes appear to retain ancestral systems for the biogenesis of their outer membranes.

The root of the bacterial tree of life remains a mystery and we do not know whether the last common ancestor of all bacteria was a monoderm or a diderm, and whether it produced endospores or not. It is reasonable to assume that the classical diderms that contain LPS have a single origin (Sutcliffe, 2010; Tocheva et al., 2016; Sutcliffe and Dover, 2016), and that they plausibly evolved via an endospore released by an ancestral monoderm (Dawes, 1981; Vollmer, 2012; Tocheva et al., 2011). And now the work of Antunes et al. suggests that most Firmicutes lineages became secondarily monodermic on multiple occasions. Is the same true for the Actinobacteria and the Chloroflexi, the other two phyla that contain monoderms? It is also noteworthy that the three monodermic phyla tend to cluster in many analyses, and are relatively close to the presumed root of the bacterial tree of life (Raymann et al., 2015; Hug et al., 2016), although resolution remains poor at the deepest phylogenetic levels. A more robust phylogenetic framework for bacteria is needed to make sense of these observations.

To better understand the large-scale evolutionary history of bacteria, we need to answer why, how and when the major structural differences among the prokaryotes (bacteria and archaea) came to be. Antunes et al. have provided some answers to the last question (and also shown that a given major structural change can happen more than once), and planted the seeds to answer the first two questions with regard to the evolution of monodermic bacteria. Future biochemical, ultrastructural and genomic characterization of novel prokaryotic lineages, such as the CPR taxa (short for candidate phyla radiation taxa; Hug et al., 2016), will provide more raw material to reconstruct the phenotypic evolution of prokaryotes. The syntheses of these data, together with a robust phylogenetic tree of the prokaryotes, will no doubt provide new insights into the major changes in cell evolution and help to clarify the nature of the last common ancestor of bacteria.

## References

[bib1] Antunes LCS, Poppleton D, Kling A, Criscuolo A, Dupuy B, Brochier-Armanet C, Beloin C, Gribaldo S (2016). Phylogenomic analysis supports the ancestral presence of LPS-outer membranes in the Firmicutes. eLife.

[bib2] Cavalier-Smith T (2006). Rooting the tree of life by transition analyses. Biology Direct.

[bib3] Dawes IW, Carlile MJ, Collins JF, Moseley BEB (1981). Sporulation in evolution. Cellular and Molecular Aspects of Microbial Evolution.

[bib4] Hug LA, Baker BJ, Anantharaman K, Brown CT, Probst AJ, Castelle CJ, Butterfield CN, Hernsdorf AW, Amano Y, Ise K, Suzuki Y, Dudek N, Relman DA, Finstad KM, Amundson R, Thomas BC, Banfield JF (2016). A new view of the tree of life. Nature Microbiology.

[bib5] Raymann K, Brochier-Armanet C, Gribaldo S (2015). The two-domain tree of life is linked to a new root for the Archaea. PNAS.

[bib6] Sutcliffe IC (2010). A phylum level perspective on bacterial cell envelope architecture. Trends in Microbiology.

[bib7] Sutcliffe IC, Dover LG (2016). Comment on "Sporulation, bacterial cell envelopes and the origin of life" by Tocheva et al. Nature Reviews Microbiology.

[bib8] Tocheva EI, Matson EG, Morris DM, Moussavi F, Leadbetter JR, Jensen GJ (2011). Peptidoglycan remodeling and conversion of an inner membrane into an outer membrane during sporulation. Cell.

[bib9] Tocheva EI, Ortega DR, Jensen GJ (2016). Sporulation, bacterial cell envelopes and the origin of life. Nature Reviews Microbiology.

[bib10] Vollmer W (2012). Bacterial outer membrane evolution via sporulation?. Nature Chemical Biology.

